# Frame vibration states identification for corn harvester based on joint improved empirical mode decomposition - Support vector machine method

**DOI:** 10.3389/fpls.2023.1065209

**Published:** 2023-03-14

**Authors:** Jun Fu, Chao Chen, Rongqiang Zhao, Zhi Chen, Dan Li, Yongliang Qiao

**Affiliations:** ^1^ College of Biological and Agricultural Engineering, Jilin University, Changchun, China; ^2^ Key Laboratory of Efficient Sowing and Harvesting Equipment, Ministry of Agriculture and Rural Affairs, Jilin University, Changchun, China; ^3^ Key Laboratory of Bionic Engineering, Ministry of Education, Jilin University, Changchun, China; ^4^ Department of Science and Technology Development, Chinese Academy of Agricultural Mechanization Sciences, Beijing, China; ^5^ College of Astronautics, Nanjing University of Aeronautics and Astronautics, Nanjing, China; ^6^ Faculty of Engineering and Information Technologies, Australian Centre for Field Robotics, University of Sydney, Sydney, NSW, Australia

**Keywords:** reliability of harvester, frame vibration, decrease noise, low order vibration, vibration frequency

## Abstract

The frame of corn harvester is prone to vibration bending and torsional deformation due to the vibration caused by field road bumps and fluctuations. It poses a serious challenge to the reliability of machinery. Therefore it is critical to explore the vibration mechanism, and to identify the vibration states under different working conditions. To address the above problem, a vibration state identification method is proposed in this paper. An improved empirical mode decomposition (EMD) algorithm was used to decrease noise for signals of high noise and non-stationary vibration in the field. The support vector machine (SVM) model was used for identification of frame vibration states under different working conditions. The results showed that: (1) an improved EMD algorithm could effectively reduce noise interference and restore the effective information of the original signal. (2) based on improved EMD – SVM method identify the vibration states of the frame with the accuracy of 99.21%. (3) The corn ears in grain tank were not sensitive to low order vibration, but had an absorption effect on high order vibration. The proposed method has the potential to be applied for accurately identifying vibration state and improving frame safety.

## 1 Introduction

Vibration is the reciprocating motion of a mechanical or structural system near its equilibrium position. It often brings many serious hazards in engineering, and is usually the direct cause of mechanical and structural damage and failure. For example, due to vibration, the machining accuracy of machine tools is reduced, maintenance costs are increased, electronic equipment cannot work properly, and vehicle frames fail and fracture (Maia et al., 2003; Bin et al., 2018; Han et al., 2020). In addition, according to statistics, about 42% of total gas turbine failures have been caused because of vibration (Meher-Homji, 1995). For corn harvesters frame, the vibration caused by field road bumps and fluctuations leads to bending and torsional deformation (Su et al., 2011), and it poses a severe challenge to the reliability of the machine.

For vibration problems, a great deal of research has been done by domestic and foreign scholars. Frequency response function (FRF) is a common method and it represents the relationship between input and output when excitation and response signal are known (Cai and Hou, 2021). Particularly, FRF was indispensable in the field of structural dynamics which studies the characteristics of vibration systems (Allemang et al., 2022). By establishing the dynamic differential equation of the cab, Li et al. calculated the FRF and optimized the vibration isolation performance (Li et al., 2013). Chen et al. solved the analytical expression of the vibration response for the pedestrian bridge. The research results showed that the dynamic response can be easily calculated by using the simplified formula for the bridge structure (Chen et al., 2018). However, the calculation of FRF needs to know the input and output at the same time to establish the relationship model of the structure.

For the case that only the output signal is known, the output-only modal identification method is widely accepted in the field of vibration. This method could directly identify modal parameters from the structural response signals (Poulimenos and Fassois, 2009; Nagarajaiah et al., 2014). Blind source separation (BSS) was a powerful signal processing tool in the 1990s (Cardoso, 1998). Compared with the parametric modeling method, the main advantages of the BSS were simple technology, high computational efficiency, nonparametric, and no prior information of dynamic system (Yu, 2019; Naghsh et al., 2022). The working mode analysis method based on vibration response transmissivity has developed rapidly due to the unique dynamic characteristics of vibration response transmissivity. Sun et al. directly estimated the vibration mode of the structure by using the characteristic that the response transfer ratio was independent of the input at the system pole (Quin et al., 2017). However, only the output mode identification method requires that the number of test sensors should be more than the number of signal sources, and the amplitude of the separated signal is unstable.

In addition to the above methods, the identification of structural modal parameters by finite element software is also a universal method in vibration research. Modal parameters are the function of structural physical characteristics. Its modal parameters (vibration mode, damping ratio and frequency) will also change when the structure is damaged or the physical parameters (mass, stiffness and damping) change. Bum et al. used first order analysis technology to design a vehicle sub-frame and proposed an equivalent model of vehicle sub-frame composed only of beam elements (Kim et al., 2009). Shrinidhi et al. used ANSYS software to analyze the ladder frame and extracted the first six non-zero natural frequencies and their corresponding modes of vibration (Rao and Bhattu, 2019). In the research of harvester vibration, Li et al. constructed the parameterized models of the harvester chassis frame and the header frame respectively, then optimized them (Li et al., 2013; Li et al., 2014). Xu et al. analyzed the vibration of the engine, header and vibrating sieve respectively, then revealed the influence of main vibration source and feeding rate ( Li et al., 2014; Gao et al., 2017). Yao et al. adopted the method of combining finite element analysis with vibration to explore the corresponding relationship between vibration dominant frequency and modal shape, and optimized the header and frame to avoid the resonance dominant frequency (Yao et al., 2015; Yao et al., 2017). Chen et al. established a 7-degrees of freedom dynamic model of the harvester frame, and revealed the law of modal shape and frequency for the frame (Chen et al., 2020). However, the above method requires more meshes to obtain better accuracy, and it leads to a large amount of calculation in the whole analysis. On the other hand, the above researches focused on the interpretation of mechanical structure model and ignored the exploration of the key information contained in vibration signal.

Hence, this paper starts from the perspective of signals to study frame vibration states of corn harvester and explore the vibration mechanism. The absorption of vibration energy by corn ear is different for every transportation condition when the grain tank is full. On the micro level, it is reflected that corn ear may hinder a certain order or several orders of the signal but is not sensitive to other orders, making the original signal become the superposition of the remaining order signals. On the macro level, the original excitation signal is distorted, and it is different from the theoretical calculation value. It will lead to abnormal vibration of the frame and even resonance, which seriously affects the reliability of the machine. The vibration signal will show the characteristics of non-stationary and high noise caused by its many parts, complex structure, and the influence of field road fluctuation and turbulence for the corn harvester. The EMD has the advantages of adaptive decomposition of noisy and non-stationary signals without considering the basis function (Kedadouche et al., 2016). It can realize the secondary filtering of signals and restore the information of original vibration signals. Therefore, the EMD is used to process signals in this paper. However, mode mixing occurs directly using this method due to the lack of a complete theoretical basis for the EMD algorithms, i.e. a separate intrinsic mode function(IMF) signal may contain different time scales, affecting the synthesis of subsequent signals and the extraction of features. So original algorithm need to be improved before using EMD decomposition signals.

Deep learning is a special type of machine learning methods capable of extracting the optimal input representation directly from the raw data without user intervention [Dong et al., 2021]. It is popular because of its strong learning ability and wide coverage. However, deep learning needs to rely on a large number of sample data in practical application. For field vibration testing, it involves a variety of working conditions and machine models, which increases the complexity of data. Once the external conditions change, data needs to be collected again. Thus, the method of deep learning is not universal enough for the problems studied in this paper. On the other hand, the model design of deep learning is very complex. If the ready-made model is used, the final results will have a big deviation, and the final results may not be explained (Xu et al., 2020). On the contrary, SVM is a small sample learning method with solid theoretical basis. In essence, it avoids the traditional process from induction to deduction, and realizes efficient “transition inference” from training samples to prediction samples, which greatly simplifies the usual classification and regression problems (Li et al., 2019). At the same time, SVM is robust to the sample set, and the final result of its output is easy to interpret. Therefore, this paper adopts SVM method to identify the vibration states of the frame.

The main work of this research as follows:

(1) For the non-stationary and high noise characteristics of field vibration signal, the improved EMD method could effectively reduce noise interference and restore the effective information of the original signal.
(2) The SVM model based on power spectrum entropy (PSE) and standard deviation (SD) is established. Optimizing model parameters, and results are compared and analyzed.
(3) The established model realizes the identification of frame vibration states for corn harvester under different working conditions.

## 2 Materials and methods

### 2.1 Experimental equipment

**Figure 1:**
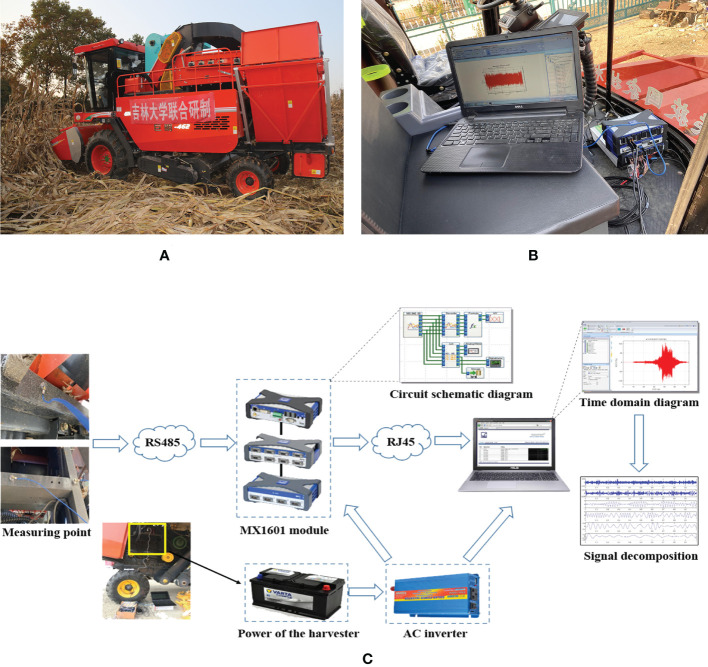
Experimental machine and wiring diagram: **(A)** Experimental corn harvester; **(B)** Test system wiring diagram; **(C)** Test system schematic diagram.

In this study, Yitong Manchu Autonomous County, Siping City, Jilin Province, located in northeast China, was selected as test area. The harvesting machine used was the self-propelled 4YZP-4Y harvester jointly developed by Jilin University and Shandong Juming Machinery Co., LTD., as shown in **Figure 1A**. MX1601 module of Hotingger Brüel & Kjær (HBK) and acceleration sensor of PCB were used in the data acquisition system. The specific parameters are shown in **Table 1**. Data test points were distributed on the harvester frame, and the wiring diagram and schematic diagram of the test instrument and equipment are shown in **Figures 1B, C**, respectively. The working conditions of experiment were that the engine runs at low speed, medium speed and high speed respectively when the grain tank was full, as shown in **Table 2**.

**Table 1:** Parameter values of the MX1601 module and acceleration sensor.

Device	Parameter	Value
MX1601 module	Bandwidth	3 kHz
	Sampling rate	20 kS/s
	Number of channels	16 channels
	Linear error	< 0.02% of full scale value
356A33 sensor	Sensitivity	1.02mv/(m/s^2^)
	Measuring range	2-10000Hz

**Table 2:** Engine speed under different working conditions.

Working conditions	S1	S2	S3
Engine speed	750r/min	1500r/min	2300r/min

### 2.2 Proposed vibration noise removal method

In this section, we first review the standard EMD algorithm, and then introduce the calculation process of the proposed algorithm in detail.

#### 2.2.1 Standard EMD framework

The EMD performed signal decomposition according to the time scale characteristics of the data itself without setting any basis function in advance (Shen et al., 1998; Huang et al., 1998). It could decompose a complex signal into a limited number of IMF components, which contained local characteristic signals of different time scales for the original signal. The EMD decomposition method is based on the following assumptions:

(1) The signal has at least two extreme points, a maximum and a minimum;
(2) The characteristic time scale is defined by the length of time between two extreme points;
(3) If the signal data lacks extremum points but there are deformation points, the extremum points can be obtained by differentiating the data once or several times, and then the decomposition results can be attained by integration.

**Figure 2:**
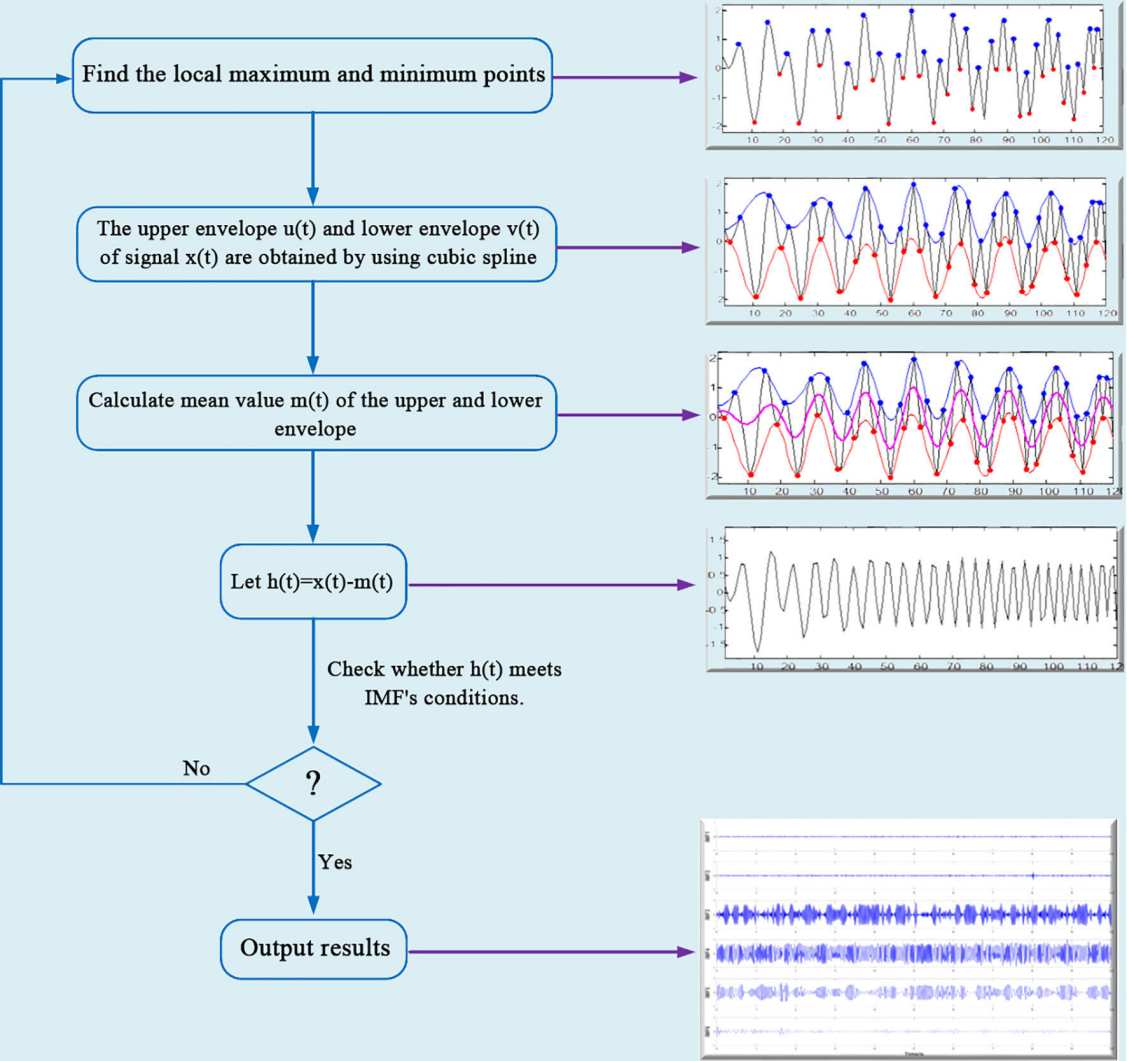
Standard EMD flow chart.

The specific decomposition process of the EMD can be divided into the following steps, as shown in **Figure 2**:

#### 2.2.2 Proposed algorithm

The main limitation of the EMD was mode mixing (Dai et al., 2019). The root cause of this phenomenon was the influence of abnormal events in the signal, such as discontinuous signal, pulse interference and noise. Based on the theory above, the proposed EMD algorithm can be divided into the following steps (*)

Step1. Applying equations (1) to (7) to obtain the Hilbert marginal spectrum of the IMF1. Specifically, it can be described as:

(1) The original signal *x(t)* is decomposed by standard EMD to obtain IMF1. Hilbert transformation is performed on the IMF1 to obtain *v(t)*, namely:


(1) $$v(t)=\frac{1}{\pi }\int_{-\infty }^{\infty }\frac{u(\tau )}{\tau -t}d\tau ,$$

(2) Construct the analytic signal:


(2) $$z_{i}(t)=u(t)+jv(t)=a_{i}(t)e^{j\varphi_{i}(t),}$$

(*) If you need the source codes for this article, please contact the author at zrq@jlu.edu.cn

(3) The amplitude and phase functions are obtained:


(3) $$a_{i}(t)=\sqrt{u_{i}^{2}(t)+v_{i}^{2}(t),}$$

(4) $$\varphi_{i}(t)=\arctan\frac{v_{i}(t)}{u_{i}(t)},$$

(4) The instantaneous frequency is further calculated as follows:


(5) $$f_{i}(t)=\frac{1}{2\pi }\cdot \omega_{i}(t)=\frac{1}{2\pi }\cdot \frac{d\varphi_{i}(t)}{dt},$$

(5) Continue to obtain Hilbert spectrum *H(w,t)*:


(6) $$H(\omega ,t)=RP\sum_{i=1}^{n}a_{i}(t)e^{j\int \varphi_{i}(t)},$$

(6) Hilbert marginal spectrum is obtained:


(7) $$h(\omega )=\int_{0}^{T}H(\omega ,t)dt,$$

In equation (6), RP represents the real part, T represents the total length of the signal in equation (7).

Step2. The analytic signal *y(t)* is constructed by equation (8) to (9). It can be described as:

(1)The average instantaneous frequency of the IMF1 can be calculated by equation (8) according to the energy mean method (Deering and Kaiser, 2005).


(8) $$\bar{f}=\frac{\sum_{i}^{k}a_{1}(i)f_{1}^{2}(i)}{\sum_{i}^{k}a_{1}(i)f_{1}(i)},$$

Where *a_1_(t)* is the Hilbert envelope amplitude of the IMF1, *f_1_(t)* is the instantaneous frequency of the IMF1.

(2)Solve the analytic signal *y(t*):


(9) $$y(t)=a_{0}\sin(2\pi ftf_{s}),$$

Where *f_s_* is the sampling rate of the signal; As a rule of thumb, *a_0_* is 1.6 times the average amplitude of the signal component.

Step3. The analytic signal *y(t)* is mixed with the original signal *x(t)* to obtain *x_1_(t)* and *x_2_(t)*:


(10) $$x_{1}(t)=x(t)+y(t),$$

(11) $$x_{2}(t)=x(t)-y(t),$$

Step4. the EMD algorithm is used to decompose *x_1_(t)* and *x_2_(t)* respectively, and the intrinsic modal functions *L_1_(t)* and *L_2_(t)* are attained. The intrinsic modal function of *x(t)* is obtained as *L(t)*.


(12) $$L(t)=\frac{L_{1}(t)+L_{2}(t)}{2},$$

### 2.3 Vibration states identification method

In statistics, the SD reflects the dispersion of a data set. In the time domain vibration signal, it could reflect the change of signal energy (Yao et al., 2010; Ji et al., 2018). The value of the SD will increase with the advance of frame vibration amplitude. The PSE was a dimensionless index, which could reflect the distribution of different frequencies in the frequency band (Cao et al., 2015). When the frequency component is widely distributed in the frequency band, the uncertainty of the distribution is high, leading to the increase of the PSE. On the contrary, when the frequency components are concentrated in a certain frequency band, the uncertainty of frequency distribution is low, resulting in the decrease of the PSE.

The SD is the root of the sum of the squares of the deviations. The exact value can be calculated by the equation (13):


(13) $$s=\sqrt{\frac{1}{n-1}\cdot \sum_{i=1}^{n}{\left(x_{i}-\bar{x}\right)}^{2}},$$

where $\bar{x}=\frac{1}{n}.\sum_{i=1}^{n}x_{i}$ , *n* is the length of the entire data *x*.

The PSE is the extension of Shannon entropy in the frequency domain, which is related to the distribution of frequency components. The calculation method of the PSE is as follows (Shen et al., 1998; Cao et al., 2015):

Step1. The power spectrum of signal *x(t)* can be attained by equation (14).


(14) $$s(f)=\frac{1}{2\pi N}{\left|X(\omega )\right|}^{2},$$

where *N* is the length of the data *x(t)*. *X(w)* denotes the Fourier transform of *x(t)* by the fast Fourier transform (FFT).

Step2. According to equation (15), the probability density function of the spectrum is estimated by normalizing all frequency components:


(15) $$p_{i}=\frac{s(f_{i})}{\sum_{k=1}^{N}s(f_{k})},$$

where *s(f_i_)* is the spectral energy of the *i-th* frequency component *f*_*i*_, *p*_*i*_ is the corresponding probability density, *N* is the total number of frequency components in FFT.

Step3. The corresponding PSE is defined as:


(16) $$H=-\sum_{k=1}^{N}p_{i}\cdot \log(p_{i}),$$

(17) $$E=\frac{H}{\log N}=\frac{-\sum_{k}^{N}p_{i}\cdot \log(p_{i})}{\log N},$$

The PSE is a dimensionless index within the range of [0,1], where 1 represents the spectrum with relatively uniform and uncertain frequency component distribution, and 0 represents the least uncertain distribution.

**Figure 3:**
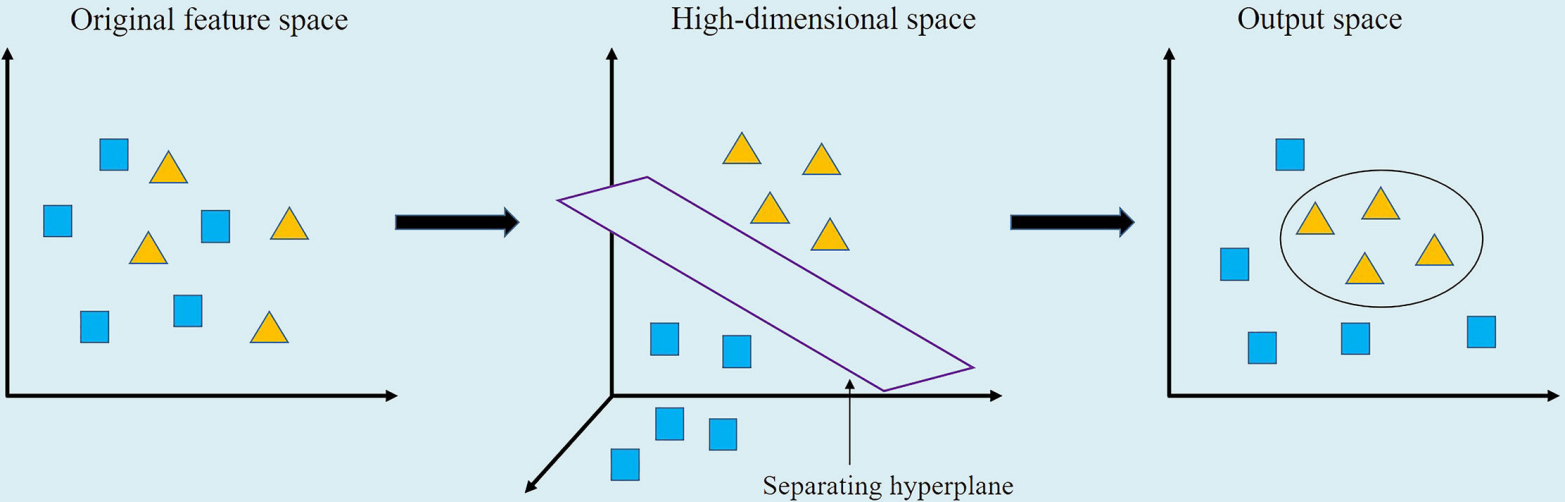
Nonlinear SVM schematic diagram.

The SVM was a kind of generalized linear classifier that classified data by supervised learning (Cortes and Vapnik, 1995). Its decision boundary was the maximum margin hyperplane to be solved for learning samples, and classification was achieved by establishing a mapping model between input feature vector and output label vector. That was, after a sample input was given, the estimated type of the corresponding output label under the mapping relationship could be obtained. The SVM established a model to convert the low-dimensional input x and output y into inner product of high-dimensional space through kernel function, as shown in **Figure 3**.

## 3 Experimental setup

The process of this study is introduced as follows. First, the original signal was decomposed after filtering to obtain a series of intrinsic mode functions (IMFs). Then, the relative energy ratio of each IMF was calculated, and several IMFs with high relative energy ratio were selected to combine into a new signal. In order to reduce the amount of calculation, the PSE and the SD were calculated for every 200 data, and 326*2 feature sets were obtained; Working conditions S1, S2, and S3 were labeled as Label1, Label2, and Label3 respectively; Secondly, a random function was used to scramble the ordinal matrix to get a number sequence again to avoid overfitting; Next, 1 to 250 rows were used as the training sets and 251 to the last rows were used as the test sets; Finally, the SVM was used to establish frequency recognition model.

**Figure 4:**
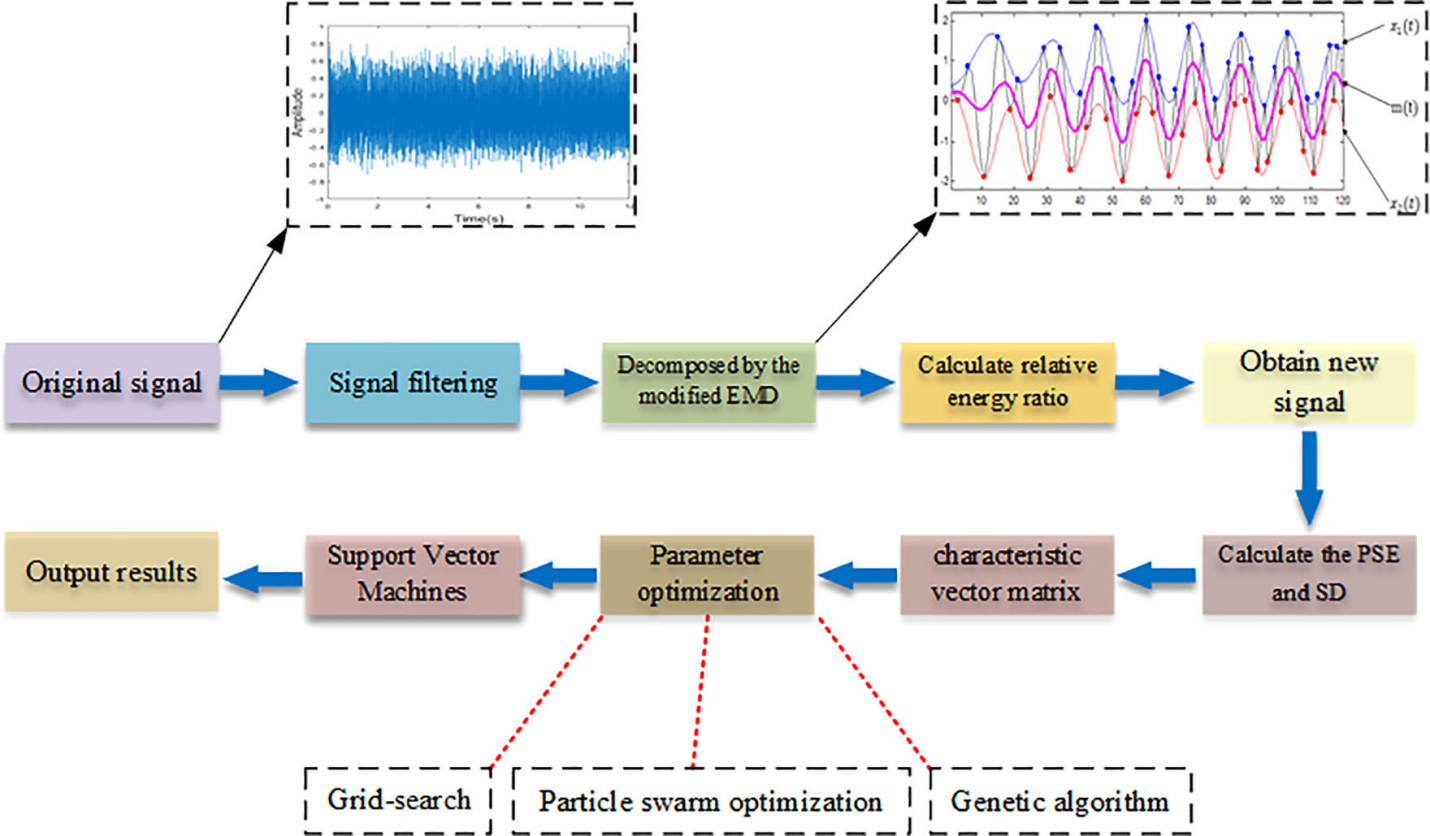
The flow chart in this study.

For the SVM model, if the parameters c and γ are too small, it may be underlearned, and vice versa, it will be overlearned. All of these situations will affect the accuracy of model training. Hence, finding appropriate parameters is particularly critical to the learning ability of the model. In this study, we used particle swarm optimization (PSO), genetic algorithm and grid search to optimize both two parameters respectively. The whole algorithm is summarized in **Algorithm 1**. The identification flow diagram is shown in **Figure 4**.

Algorithm 1 Proposed algorithm.
**Input:**
Original signal matrix *AA*, sampling frequency *f_s_
*, empty column vector *y*, threshold *ϵ*.
1:
*IMF*←emd(*AA*)
2:
Find the number of columns of *IMF* and label it *n*
3:
**for** 1 to *n*
4:
*x*←*IMF*(*i*),:
5:
Solve (3) to obtain amplitude *A_m_
*
6:
*A*←1.6*mean(*Am*)
7:
Obtain *L_i_(t)* by (9) to (12)
8:
Calculate the relative energy ratio of each *L_i_(t)*, denoted as *E_i_
*
9: **if** *Ei*>*ϵ* **then**
10:  *y*←*y*+ *L_i_(t)*
11: **end if**
12: **end for**
13: Calculate *PSE* and *SD* of *y* using equations (13) to (17)
14: Construct the eigenvector matrix, optimize the *SVM* model
**Output:** Predicted and true value

## 4 Results and discussion

### 4.1 Signal decomposition and construction

**Figure 5:**
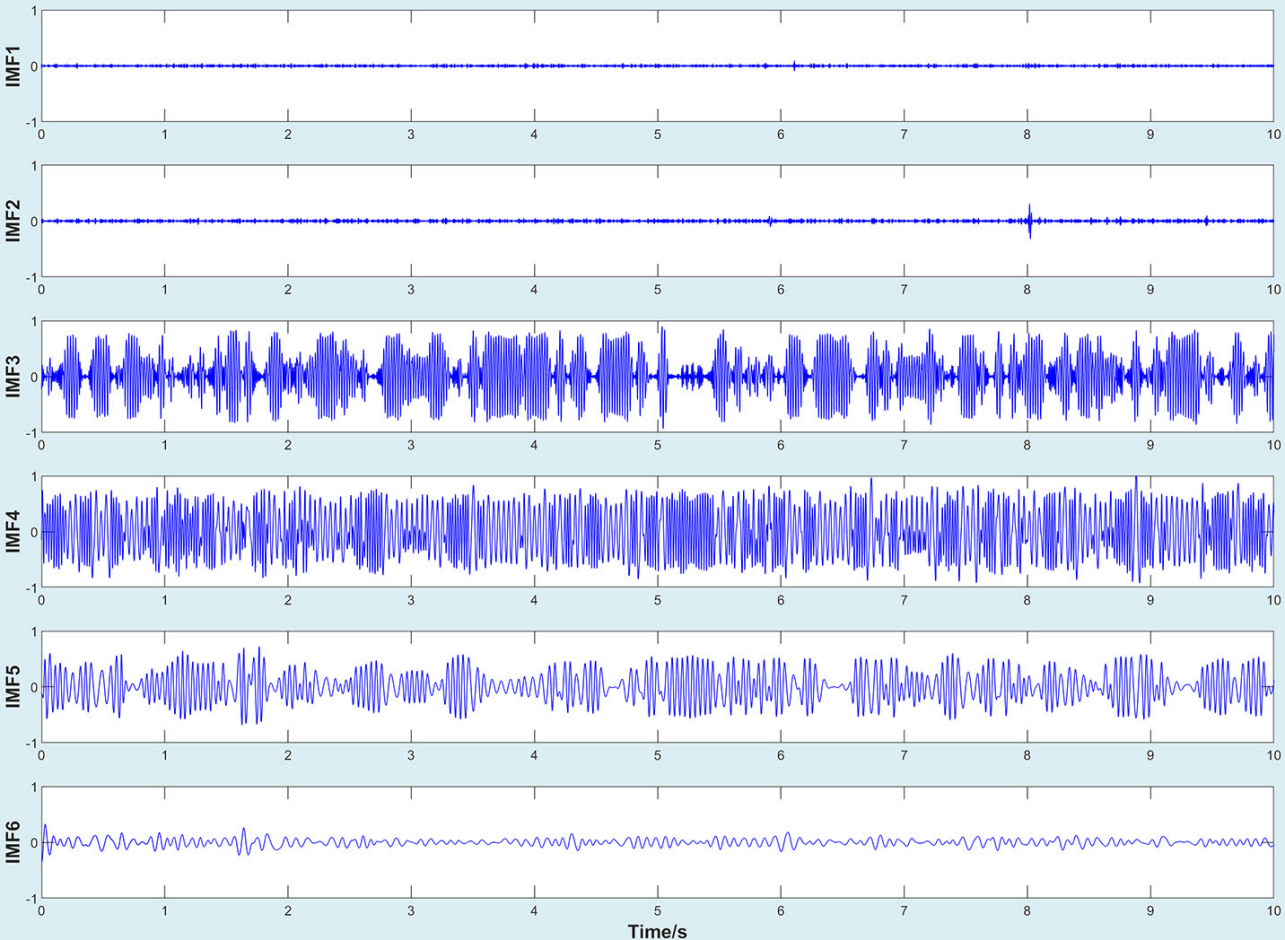
IMF1-IMF6.

**Figure 6:**
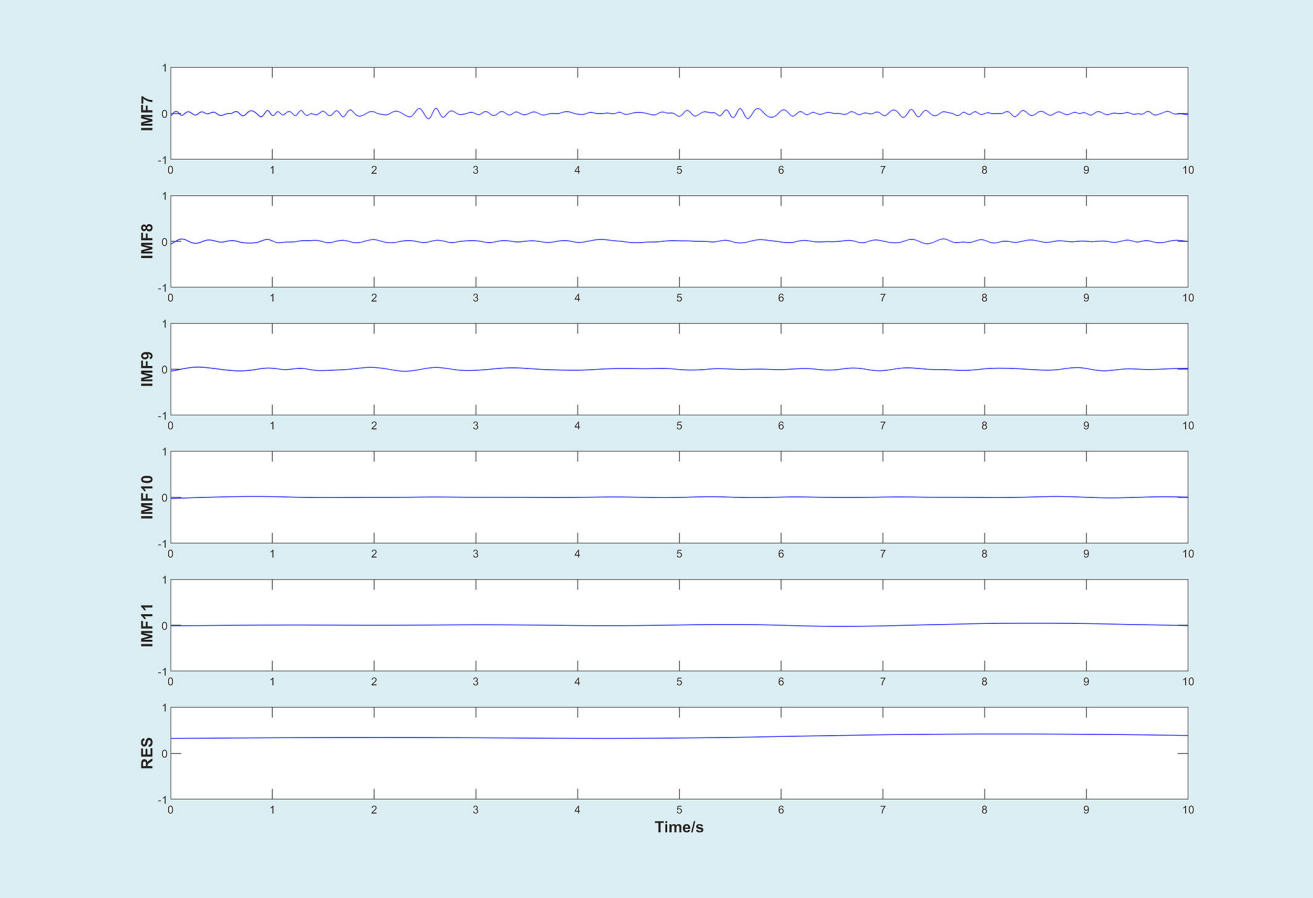
IMF7-IMF12.

After filtering, the decomposition of the original signal by improved EMD algorithm would generate a series of intrinsic modal functions and a residual signal, as shown in **Figures 5**, **6** (Take working condition S1 as an example).

According to **Figures 5**, **6**, IMFs were arranged from high frequency to low frequency. The vibration amplitudes of IMF3, IMF4, and IMF5 were larger than that of other IMFs, which indicated that the vibration of the original signal was mainly the superposition of these three vibration components. On the other hand, by observing the images of IMF3, IMF4, and IMF5, it could be seen that the waveform showed regular periodic changes. Thus, it could be inferred that the excitation of a single component in the original signal also changed periodically. In addition, compared with standard EMD, the improved EMD algorithm could not completely eliminate mode mixing, but could reduce its interference to a certain extent.

**Figure 7:**
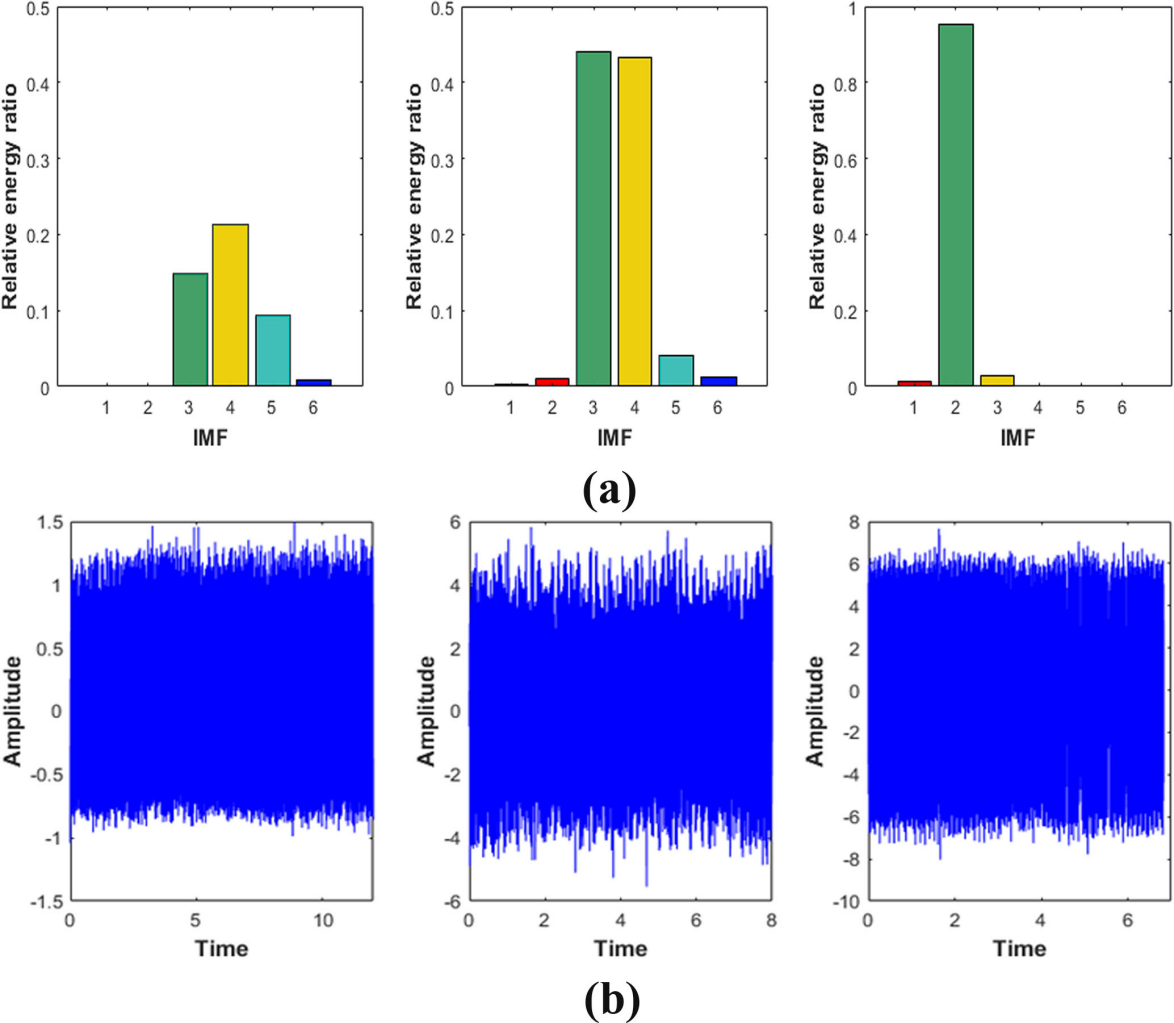
**(A)**.Relative energy ratio of the first six IMFs under Condition S1 Condition S2 Condition S3,respectively. **(B)**.New signal based on the relative energy ratio.

For the purpose of reconstructing the essential signals, IMFs need to be selected for this work. IMFs with high relative energy ratio contained most of information for original signal (Li et al., 2007; Ji et al., 2018). Therefore, we calculated the relative energy ratio of decomposed signals to extract valuable components, as shown in **Figure 7A**. It could be seen that the relative energy ratios of the IMF3, IMF4, and IMF5 were much higher than that of other IMFs, which was consistent with the above analysis. Thus, IMF3, IMF4, and IMF5 were selected to be reconstructed as new signals. The new signal is shown in **Figure 7B**.

### 4.2 Characteristic data analysis

**Figure 8:**
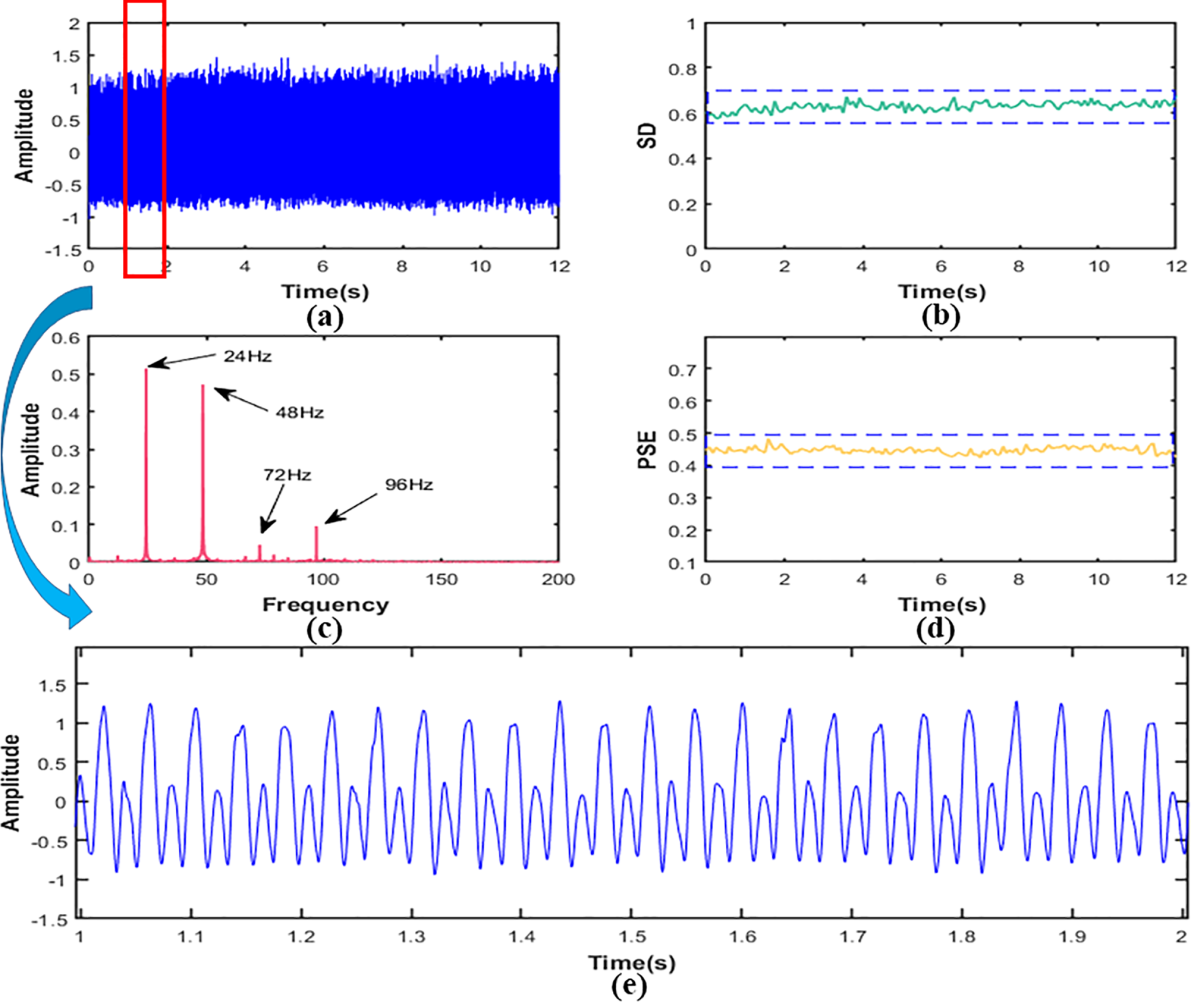
In working condition S1, signal **(A)** time domain figure,**(B)** the SD,**(C)** the PSE,**(D)** the FFT,**(E)** local amplification figure.

**Figure 9:**
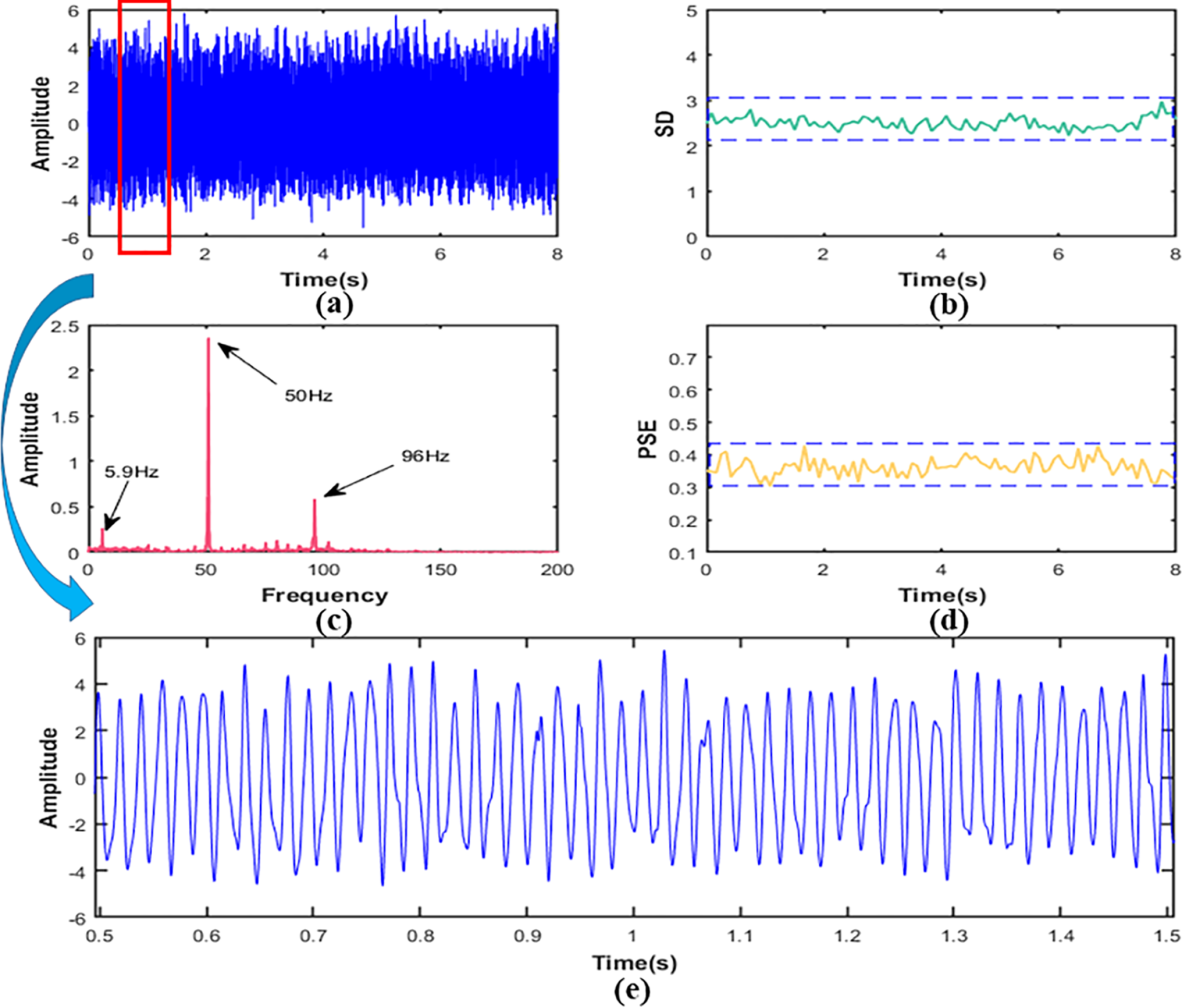
In working condition S2, signal **(A)** time domain figure, **(B)** the SD, **(C)** the PSE,**(D)** the FFT,**(E)** local amplification figure.

In this section, we drew images of reconstructed signal, the SD, and the PSE under working conditions S1, S2 and S3 respectively, as shown in **Figures 8**, **9**. In addition, in order to explain the change of frequency components more directly, we also performed local amplification and FFT on the original signal.

**Figure 8B** showed that the SD value of the signal was about 0.63 and the amplitude was relatively low. It could be indicated that the vibration energy at the measured point was low in the working condition S1. The FFT of the signal was shown in **Figure 8C**. It can be seen that the frequency components of the signal were 24Hz, 48Hz, 72Hz, and 96Hz, and the main frequency were 24Hz and 48Hz, which are about 5-10 times larger than 72Hz and 96Hz. It suggested that the vibration at the measured point was mainly transmitted from the engine. The frequency component was the ignition frequency of each order of the engine. In addition, it could be seen that the signal fluctuated greatly from the morphological characteristics of the signal in **Figure 8E**, which indicated that the signal contained many components. Looking further at **Figure 8D**, we can see that the PSE value of the signal was about 0.45.

**Figure 10:**
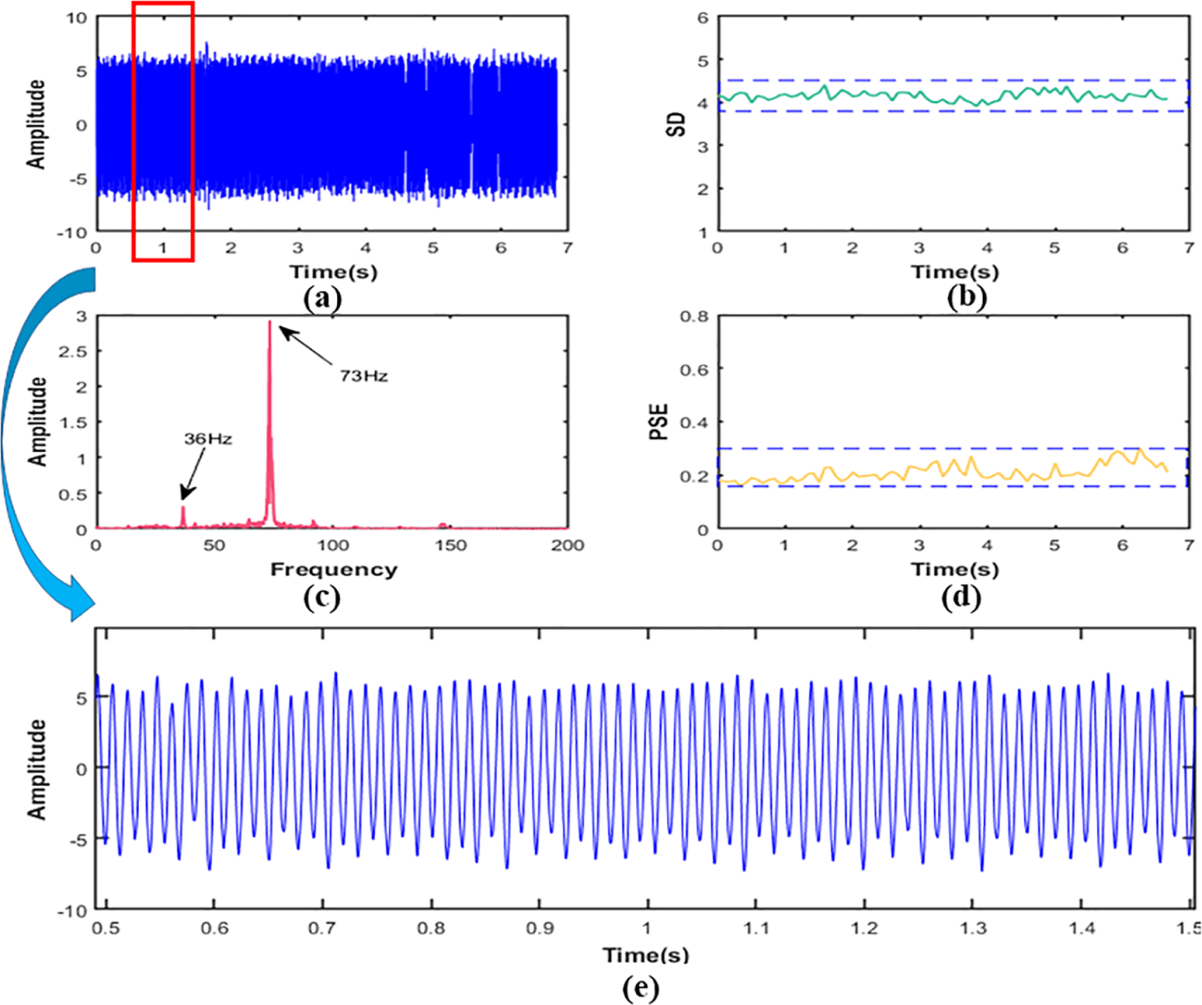
In working condition S3, signal **(A)** time domain figure, **(B)** the SD,**(C)** the PSE,**(D)** the FFT,**(E)** local amplification figure.

It was obvious from **Figure 9B** that the SD value of signal in the working condition S2 was about 2.51, and the vibration amplitude increased by about 298% compared with working condition S1, indicating that the vibration was more intense than S1. Then we calculated FFT and the results are shown in **Figure 9C**. It can be seen that vibration frequency components at the measurement point were mainly 50Hz and 96Hz, and 50Hz was the main component, and its peak value was about 4 times of 96Hz, which revealed that the vibration was mainly the first-order combustion frequency of the engine. In addition, a component of 5.9Hz could be seen in the signal from the **Figure 10C**, showing that there was not only excitation from the engine but also low-frequency excitation from the road at the measurement point, which was consistent with the research of Yao et al. (Yao et al., 2017). The PSE of the signal was shown in **Figure 9D**. Compared with 0.45 of condition S1, the value of the PSE decreased by 20% to 0.36, which was caused by the reduction of frequency component. Accordingly, the signal curve was smoother than S1, as shown in **Figure 9E**.

In working condition S3, it was clear from **Figure 10B** that the SD value of the signal was 4.15. Compared with working conditions S1 and S2, the amplitude increased by about 559% and 65% respectively. This means that the vibration at the frame was more severe than the first two working conditions and the signal contained more energy. According to **Figure 10C**, the frequency components of working condition S3 were mainly 36Hz and 73Hz, and they were frequency doubling relationship. It shows that the excitation source at the measurement point was still the engine. In addition, since the frequency division of the engine was very close to the second-order natural frequency (36.19Hz) (Yao et al., 2015), the second-order resonance of the frame might have occurred. As the frequency component in this condition was less than that in both S1 and S2, it can be seen from **Figure 9D** that the value of PSE decreased to 0.21, about 53% and 42% lower than the first two working conditions, respectively. The corresponding signal curve was also smoother, as shown in **Figure 9E**.

### 4.3 Comparative analysis of identification results based on the SVM

**Figure 11:**
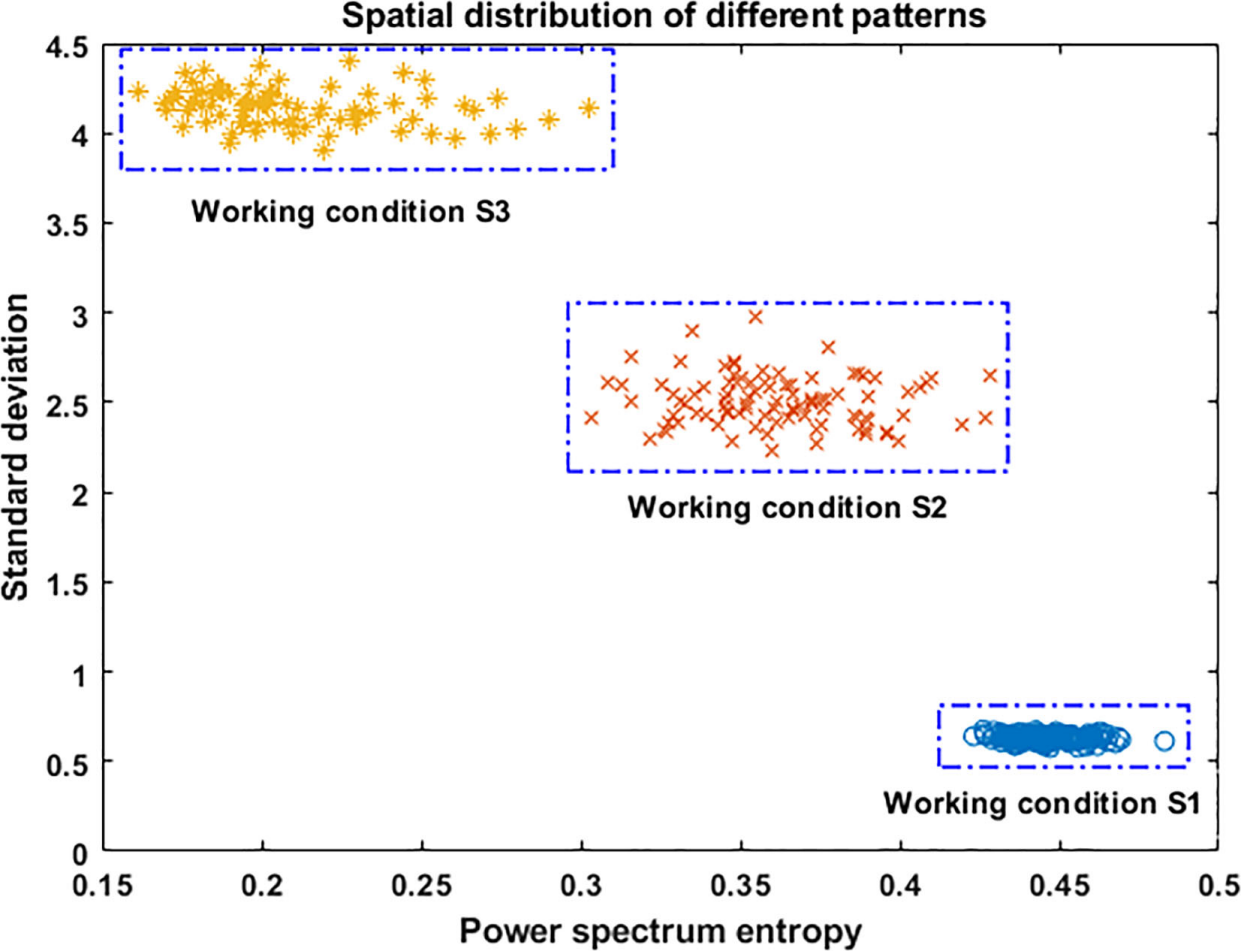
Distribution of the SD and the PSE coordinates.

From the analysis in Section 4.2, we known that the energy and frequency components of the vibration signal were different when the frame was under the three working conditions. It was directly reflected in the changes of the value of SD and the PSE. Its coordinated distribution is shown in **Figure 11**. As can be seen from **Figure 11**, the vibration energy of the frame was low and the frequency distribution was wide when the harvester was in working condition S1. The combination coordinate points of characteristic values were densely distributed in the lower right corner of the coordinate graph. The frequency component of the frame decreased and the vibration energy increased when the harvester was in working condition S2, which made the combined coordinate points of characteristic values distributed in the middle of the coordinate graph. The frequency component was further reduced, and the vibration energy was further increased when the harvester was in working condition S3. It caused the combined coordinate points of the feature values to be distributed in the upper left corner of the coordinate graph. Hence, the combination of the two characteristic values could effectively distinguish the vibration states changes in different working conditions.

Based on the above analysis, the SVM models based on the SD and the PSE were established. For illustrating the influence of the SVM model with different parameters on the recognition accuracy, we used particle swarm optimization algorithm, genetic algorithm, and grid search method to optimize the SVM model respectively. The optimal parameters and accuracy of different algorithms were shown in **Table 3**. As can be seen from **Table 3**, the test accuracy was 99.21%, 98.68%, and 97.37%, respectively, when three different algorithms were used to optimize parameters. Therefore, the accuracy of particle swarm optimization algorithm was the highest. The value of penalty parameter c was 1, and the value of kernel function parameter γ was 0.1.

**Table 3:** Optimal parameters and test accuracy by using different algorithms.

parameter optimization algorithm	optimal parameter	training accuracy	test accuracy
particle swarm optimization	c=1 g=0.1	100%	99.21%
genetic algorithm	c=0.02 g=1.14	100%	98.68%
grid search	c=2.31 g=1.83	100%	97.37%

### 4.4 Discussion on generalisation capabilities

In order to illustrate the generalisation capabilities of the algorithm proposed in this paper, this section describes the applicability of the algorithm from different optimization methods, different machines and different crops.

#### 4.4.1 Generalisation of different optimization methods

**Figure 12:**
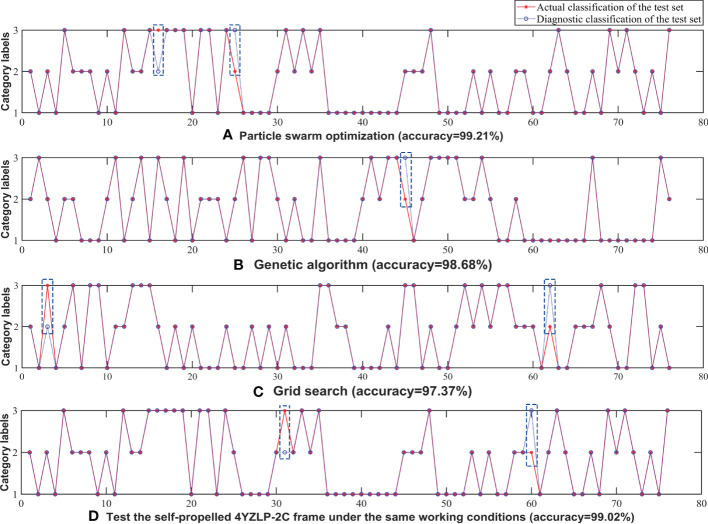
Test actual and predicted classification of data sample sets: **(A)** Particle swarm optimization (accuracy=99.21%); **(B)** Genetic algorithm (accuracy=98.68%); **(C)** Grid search (accuracy=97.37%); **(D)** Test the self-propelled 4YZLP-2C frame under the same working conditions (accuracy-99.02%).

When using particle swarm optimization algorithm, genetic algorithm and grid search method to optimize the SVM model, the actual and predicted data obtained were shown in **Figures 12A–C**.

As shown in **Figure 12A**, two samples were wrongly classified when particle swarm optimization algorithm was used to search for optimal parameters. One S3 sample was wrongly classified as S2 and the other S2 sample was classified as S3. The reason for this phenomenon, on the one hand, the discrete optimization problem was not handled well due to the characteristics of the PSO algorithm (Bratton and Kennedy, 2007), resulting in local optimal. On the other hand, no matter the frequency component or energy of the vibration signal when the harvest was in working condition S2 and S3, the difference of the characteristic values calculated was much smaller than that in S1 (the PSE difference was 42%, the SD difference was 65%), therefore it was easy to misidentify the measurement points of S2 and S3.

When genetic algorithm was applied to parameter optimization, the S2 sample was incorrectly classified as S3, as shown in **Figure 12B**. Compared with the PSO algorithm, the accuracy of genetic algorithm was reduced. The main reason was that the length of the original vibration signals was inconsistent, and it led to the different length of the calculated characteristic values. Thus the accuracy of the PSO algorithm was lower than that of the genetic algorithm.

As can be seen from **Figure 12C**, one S3 sample was inaccurately classified as S2 and the other sample was classified as S3 when the grid search method was applied. The reason for this phenomenon might be that the penalty parameter c was too large when using the grid search method, which led to the over-fitting phenomenon, and the points of S2 and S3 were confused. In addition, the higher-order signal components were very close when the harvester was in working condition S2 and S3. It made the absorption of higher-order vibration energy by corn ear tend to be consistent, resulting in a small difference in the characteristic values of the two working conditions.

#### 4.4.2 Generalisation of different machines

We also used the self-propelled 4YZLP-2C harvester to carry out the frame vibration test under the same working condition in this paper. PSE and SD were calculated after the obtained data was denoised, decomposed and reconstituted into a new signal, and then the feature vector matrix was imported into SVM and optimized by PSO. The recognition result was shown in **Figure 12D**. As shown in **Figure 12D**, two samples were wrongly classified when the self-propelled 4YZLP-2C harvester was used for testing. One S3 sample was wrongly classified as S2 and the other S2 sample was classified as S3. The reasons for this phenomenon were similar to the test of self-propelled 4YZP-4Y harvester. On the one hand, the characteristics of PSO algorithm led to local optimality; On the other hand, the difference of the feature values calculated in S2 and S3 working conditions was much smaller than that in S1 working conditions, thus it was easy to misidentify. Compared with the test of the self-propelled 4YZP-4Y harvester, the accuracy at this time is reduced by 0.19% to 99.02%, which indicates that the total error is within a reasonable range when the same method is applied to different machines.

#### 4.4.3 Application in rice and wheat harvester

The rice and wheat combine harvester is similar to the self-propelled corn harvester in the main structure. It is composed of an engine, a header, a frame, etc., and their field operation environment is very similar, resulting in vibration bending and torsional deformation of the frame. Thus, the vibration states identification method proposed in this paper also has certain applicability to the rice-wheat combine harvester.

### 4.5 Discussion

In this paper, the improved EMD algorithm combined with the SVM was used to identify the vibration state changes of the frame under different working conditions. For the corn harvester, the measured vibration signal presented high noise and non-stationary characteristics caused by the vibration of field road bumps and fluctuations when walking in the field. If the characteristic values were directly extracted for recognition, the accuracy would be affected. Therefore, the EMD method was adopted to process the signals first. However, due to the phenomenon of mode mixing in the EMD algorithm, the original algorithm was improved to enhance the accuracy of decomposition before signal processing. Secondly, we decomposed the original vibration acceleration signal to obtain a series of IMFs, and selected several groups of IMFs according to the relative energy ratio to synthesize the new signal. Compared with the original signal, the new signal at this time has greatly reduced the interference of non-stationary and high-frequency noise, therefore it can better reflect the original vibration state of the harvester. Then we calculated the PSE and SD values of the new signal, and obtained the energy variation and frequency distribution of vibration under different working conditions. According to the combination of energy change and frequency distribution, we have analyzed the main excitation source and the change of vibration order under each condition. Finally, the feature vector matrix based on PSE and SD was constructed, and the model parameters of SVM were optimized. Through comparative analysis, the recognition accuracy of these three optimization methods is 99.21%, 98.68% and 97.37%, respectively.

We could see that most of the signal information was contained in the IMF3, IMF4, and IMF5 after the original signal was decomposed when the machine was in the working condition S1. From these three signal patterns we could see that the vibration form was regular periodic vibration. As can be seen from frequency domain analysis, the components in the signal were only low frequency excitations from the engine, and the first and second order vibration was the main component. The frequency components of each order were far away from the natural frequency of the frame. In addition, the value of the SD was very small compared with S2 and S3, thus the overall vibration was relatively stable and the reliability was high. Most of the signal information was contained in the IMF3 and IMF4 when the machine was working condition S2, where the frequency components were dominated by the engine first-order ignition frequency and the low frequency excitation of the road. The main reason for this phenomenon was that as the engine speed increased, so did its higher frequency component, and it was easier to absorb by the ears in the grain tank. Hence, it could be seen from the spectrum that the frequency component of the higher order was much smaller than that of the lower order. Most of the signal information was contained in the IMF2 when the machine was in working condition S3. Similar to working condition S1, the vibration form at the frame was regular periodic vibration. With the increase of engine speed, the absorption of energy by the corn ear did not change much. In addition, the frequency division of the engine was very close to the second-order natural frequency of the frame (Yao et al., 2015), so resonance might have occurred in working condition S3. If the machine works in condition S3 for a long time, its service life will be seriously reduced. Therefore, it should be considered to change the transmission ratio or optimize the structure of the frame to enhance the reliability of the machine. Compared with the traditional analysis methods, the method proposed in this paper does not require the placement of a large number of sensors and complex numerical calculations, and only needs to obtain response datas to build the recognition model, simple operation, and high accuracy of the recognition of vibration states. This paper provides a new research idea for the vibration analysis of corn harvesters.

## 5 Conclusions

This paper proposed a novel vibration states identification method based on the improved emd and the SVM. Following conclusions can be drawn from the results of proposed methods:

(1) The improved emd algorithm can effectively reduce noise interference and restore the effective information of original signal. IMFs generated by decomposition can better reflect the change of the single component for the original excitation signal.
(2) The corn ear in the grain tank can absorb the vibration of the frame, and the absorption of vibration signal varies with the order. It reflected that it is not sensitive to the lower order signal components, but has a strong absorption effect on the higher order signal components.
(3) The proposed method can accurately identify the vibration states of the frame, and its accuracy can reach 99.21%.

However, there are still some limitations in this study: Firstly, the improved EMD algorithm still has mode mixing, and it may make IMFs lose its physical meaning and is not conducive to the later signal reconstruction. Secondly, the purpose of this study was to explore the vibration mechanism for the frame during the grain tank of corn harvester was in full load state, so other working conditions were not involved. Finally, compared with the traditional finite element analysis method, this paper did not concern the calculation of vibration mode and the damping ratio of structure. Thus, we will use mask signal method to reduce mode aliasing as much as possible in the future research. At the same time, the diversity of working conditions should be increased. Finally, we will consider identifying the parameters of the structure only from the output vibration response signal.

## Data availability statement

The original contributions presented in the study are included in the article/supplementary material, further inquiries can be directed to the corresponding author.

## Author contributions

JF: conceptualization, methodology, data curation, and writing—original draft preparation. RZ: conceptualization, resources, writing—review and editing, funding acquisition, and formal analysis. CC: resources, methodology, software, and project administration. ZC and DL: validation, data curation, writing—review and editing, and project administration. YQ: formal analysis and proofreading. All authors contributed to the article and approved the submitted version.
